# Supplementary figures and images for: Distorted TCR repertoires define multisystem inflammatory syndrome in children
Source: PLoS One. 2022 Oct 27;17(10):e0274289. doi: 10.1371/journal.pone.0274289 (PMC9612519; doi:10.1371/journal.pone.0274289)

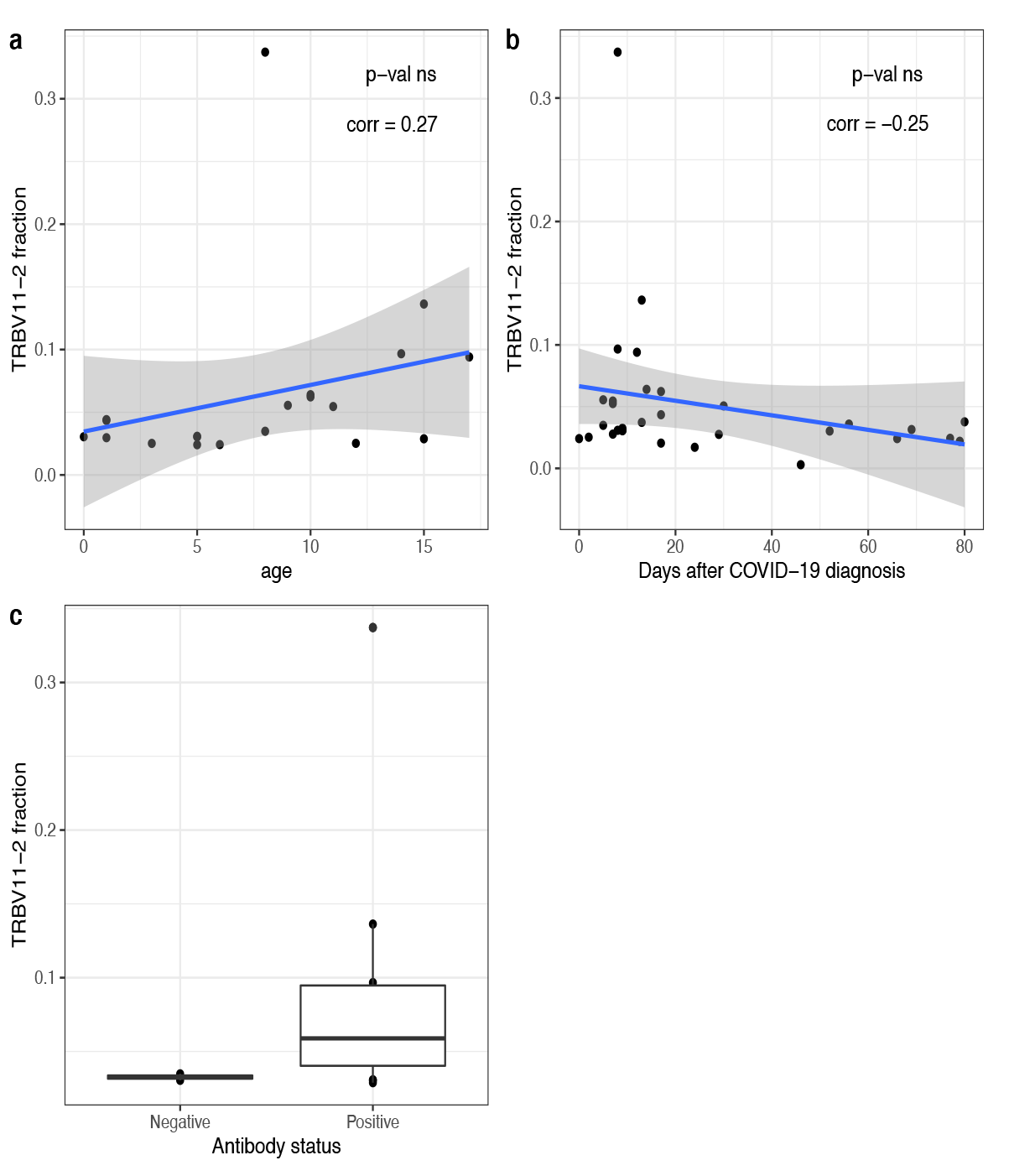

Supplement: S1 Fig — (a) The fraction of TRBV11-2 chains in patient repertoires as a function of the patients’ age with Spearman’s Rank Correlation coefficient (ρ) and P value. (b) The fraction of TRBV11-2 chains in patient repertoires as a function of the number of days after COVID-19 diagnosis with Spearman’s Rank Correlation coefficient (ρ) and P value. (c) The fraction of TRBV11-2 chains in the repertoires of antibody negative and positive patients. Significance determined by unpaired Wilcoxon test between each paediatric group, with adjustment for multiple comparisons using Benjamini-Hochberg correction, indicated by: * p<0.05, ** p<0.01, and *** p<0.001. Lack of notation for specified comparisons indicates no statistical significance. (TIF) [file pone.0274289.s002.tif]

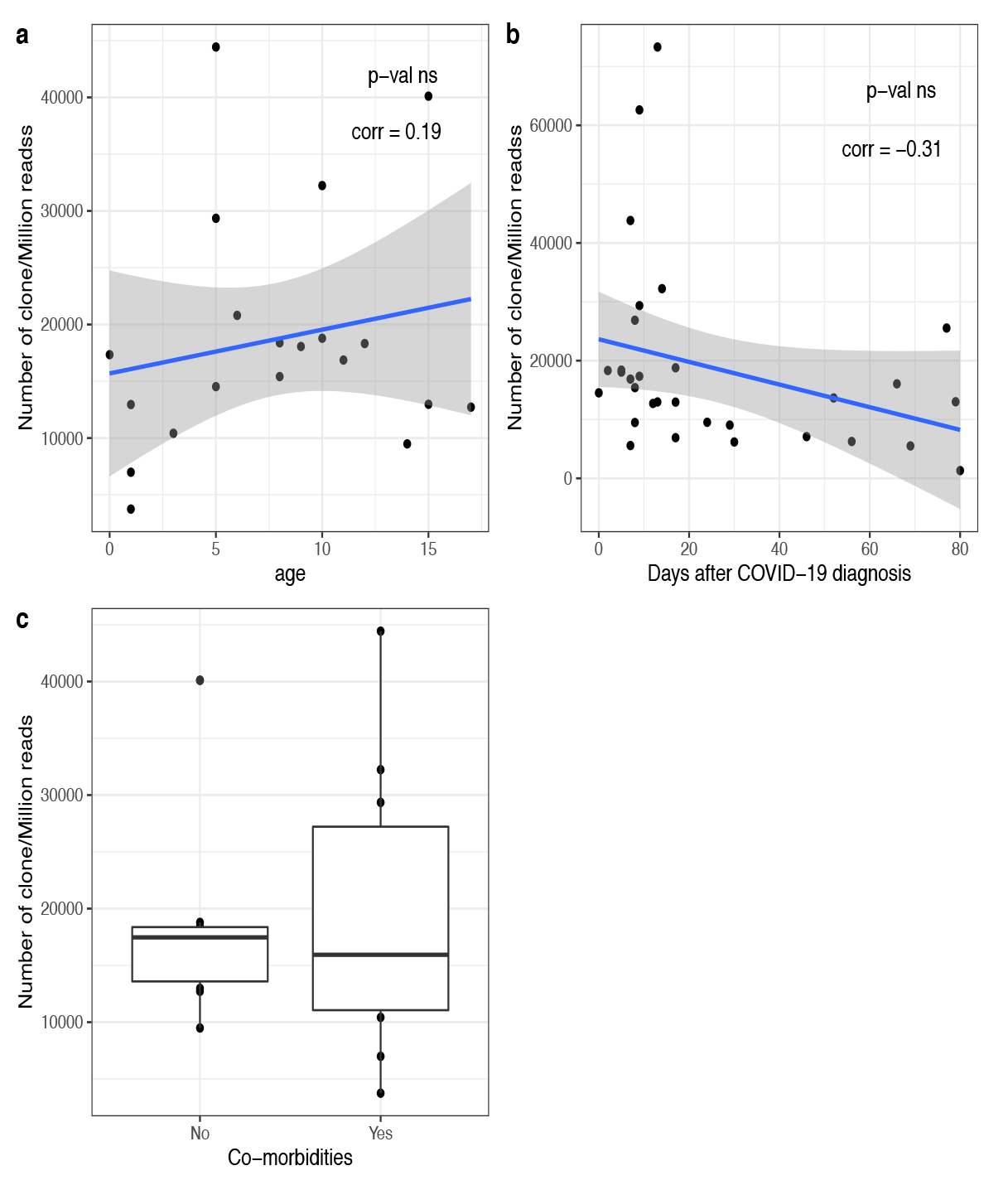

Supplement: S2 Fig — (a) The number of clones/million reads in a repertoire as a function of the patients’ age with Spearman’s Rank Correlation coefficient (ρ) and P value. (b) The number of clones/million reads in a repertoire as a function of the number of days after COVID-19 diagnosis with Spearman’s Rank Correlation coefficient (ρ) and P value. (c) The number of clones/million reads in the repertoires of patients with or without co-morbidities. Significance determined by unpaired Wilcoxon test between each paediatric group, with adjustment for multiple comparisons using Benjamini-Hochberg correction, indicated by: * p<0.05, ** p<0.01, and *** p<0.001. Lack of notation for specified comparisons indicates no statistical significance. (TIF) [file pone.0274289.s003.tif]

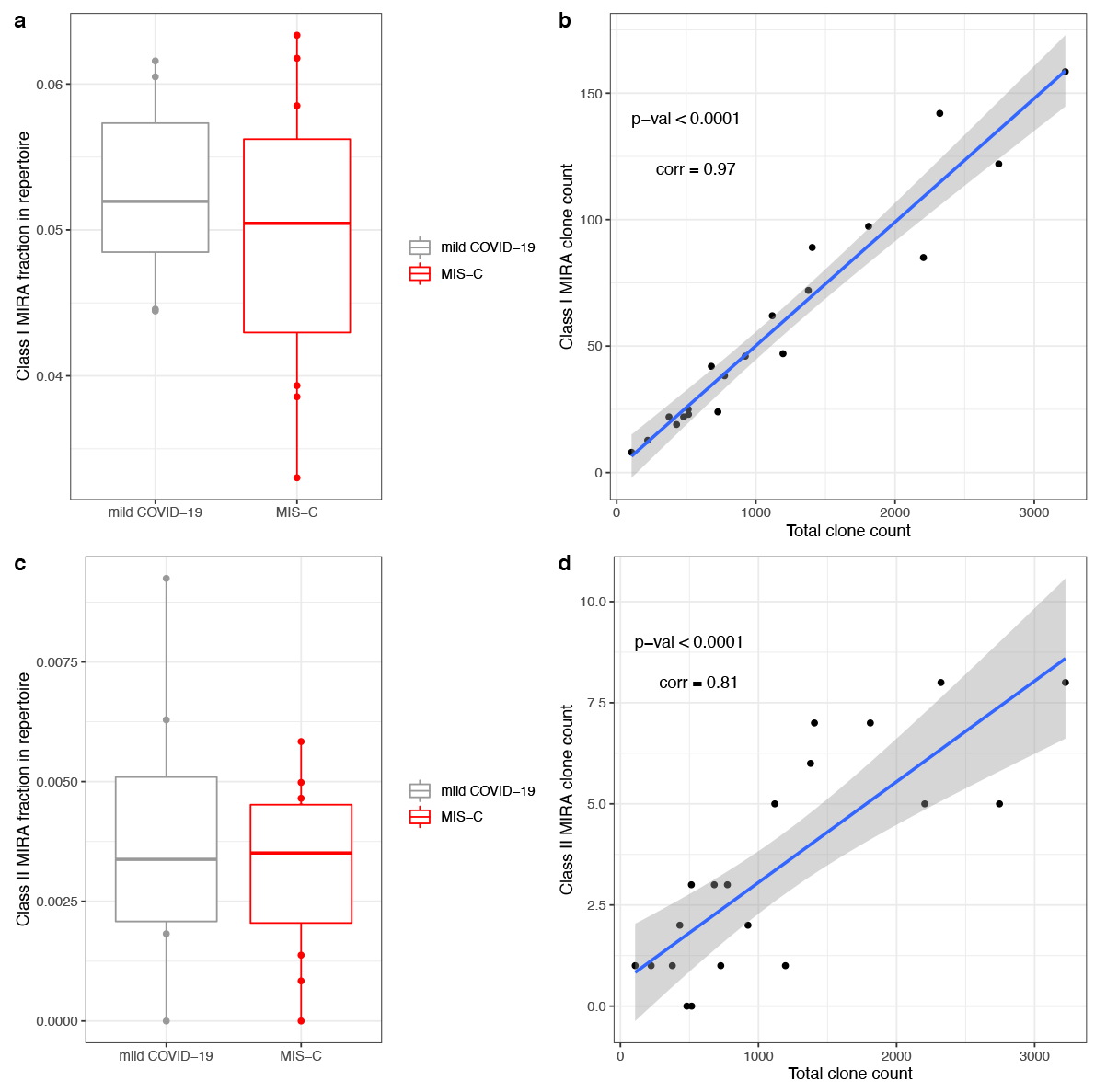

Supplement: S3 Fig — (a) The fraction of class I MIRA clones in the repertoires of patient cohorts. Significance determined by unpaired Wilcoxon test between each paediatric group, with adjustment for multiple comparisons using Benjamini-Hochberg correction, indicated by: * p<0.05, ** p<0.01, and *** p<0.001. Lack of notation for specified comparisons indicates no statistical significance. (b) The number of class I MIRA clones as the function of the total number of clones in the patient repertoires with Spearman’s Rank Correlation coefficient (ρ) and P value. (c) The fraction of class II MIRA clones in the repertoires of patient cohorts. Significance determined by unpaired Wilcoxon test between each paediatric group, with adjustment for multiple comparisons using Benjamini-Hochberg correction, indicated by: * p<0.05, ** p<0.01, and *** p<0.001. Lack of notation for specified comparisons indicates no statistical significance. (d) The number of class II MIRA clones as the function of the total number of clones in the patient repertoires with Spearman’s Rank Correlation coefficient (ρ) and P value. (TIF) [file pone.0274289.s004.tif]

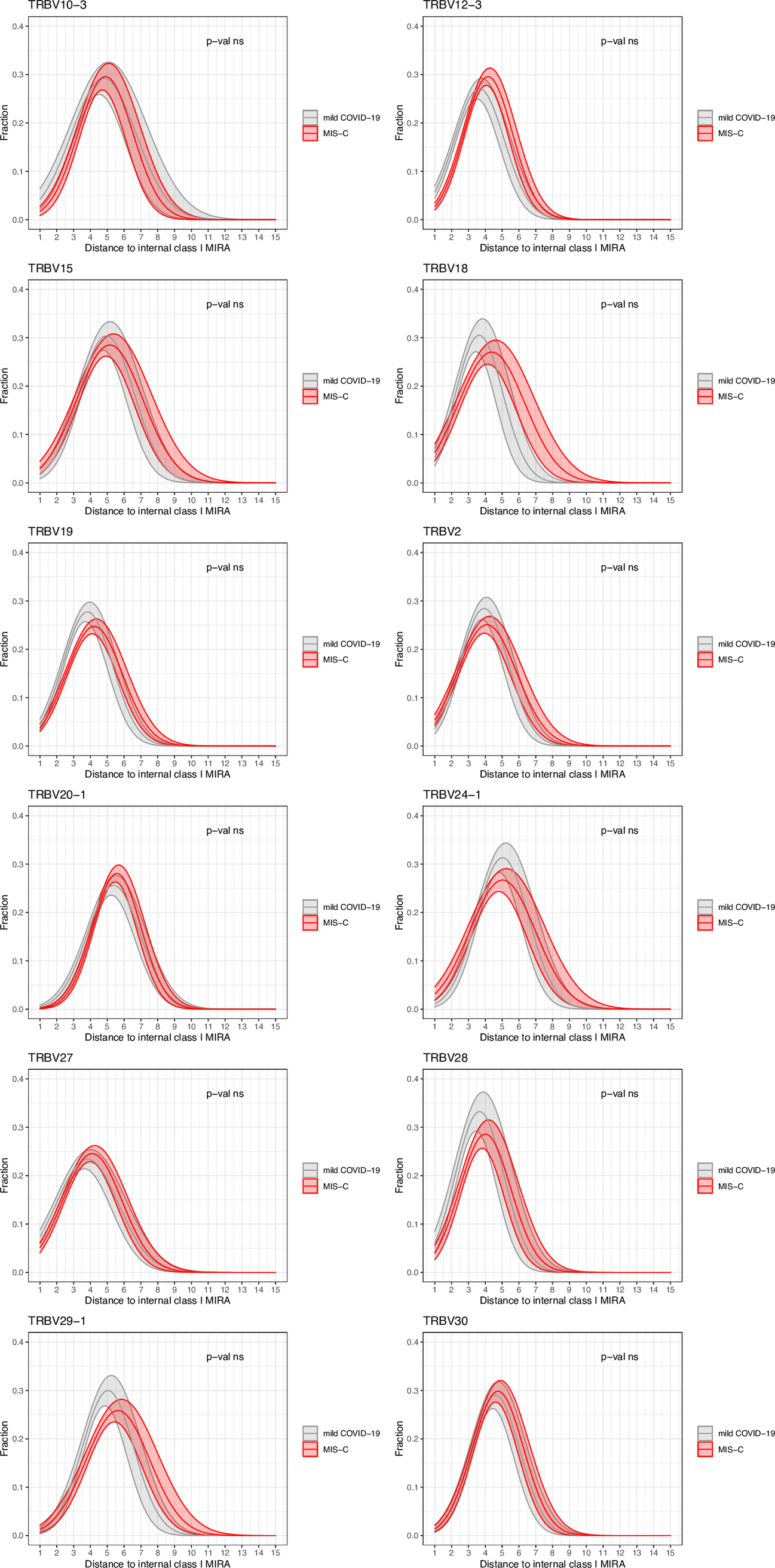

Supplement: S4 Fig — Distance to class I MIRA distribution of clones in the repertoires of children with mild or MIS-C. Distance to class I MIRA distributions were fitted with Gaussian probability density functions and the difference between patient cohorts was assessed by comparing a model fitting all symptom groups together to a model taking into account the differences between symptom groups. The difference between the two models was determined with ANOVA analysis, with all p-values corrected for multiple hypothesis testing using Benjamini-Hochberg adjustment. Solid curves show the probability distribution functions determined by fitting the patient cohorts separately, shaded areas show 75% confidence intervals. P-values denote the statistical assessment of the models fitting all patient cohorts together versus fitting each patient cohort separately. See the distance to class I MIRA distribution of TRBV11-2 clones in Fig 3b. (TIF) [file pone.0274289.s005.tif]

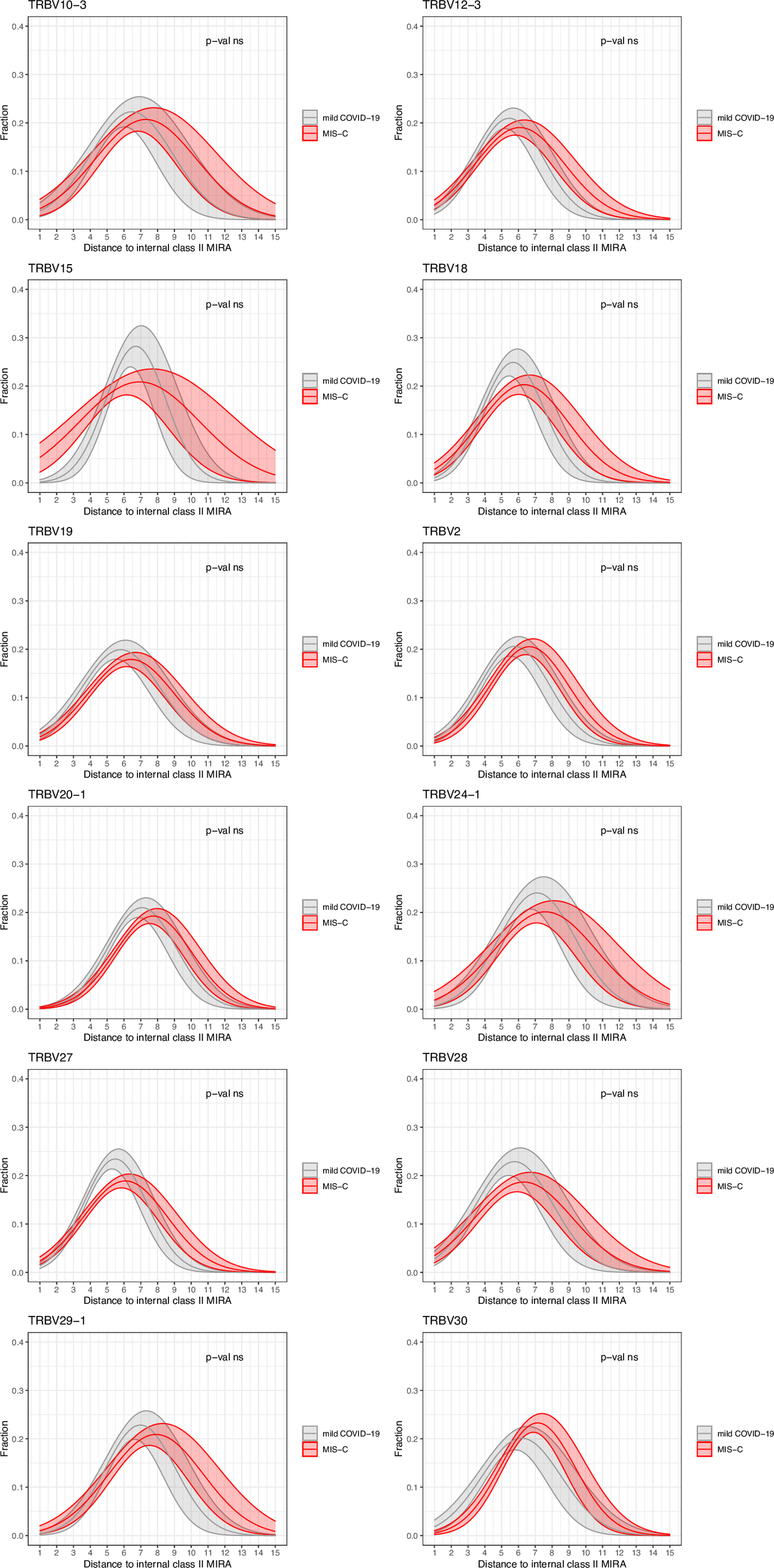

Supplement: S5 Fig — Distance to class II MIRA distribution of clones in the repertoires of children with mild or MIS-C. Distance to class II MIRA distributions were fitted with Gaussian probability density functions and the difference between patient cohorts was assessed by comparing a model fitting all symptom groups together to a model taking into account the differences between symptom groups. The difference between the two models was determined with ANOVA analysis, with all p-values corrected for multiple hypothesis testing using Benjamini-Hochberg adjustment. Solid curves show the probability distribution functions determined by fitting the patient cohorts separately, shaded areas show 75% confidence intervals. P-values denote the statistical assessment of the models fitting all patient cohorts together versus fitting each patient cohort separately. See the distance to class II MIRA distribution of TRBV11-2 clones in Fig 3c. (TIF) [file pone.0274289.s006.tif]
