# Supplementary material for: Distorted TCR repertoires define multisystem inflammatory syndrome in children
Source: PLoS One. 2022 Oct 27;17(10):e0274289. doi: 10.1371/journal.pone.0274289 (PMC9612519; doi:10.1371/journal.pone.0274289)
Supplement: Attachment — Submitted filename: Response to Reviewers.docx [file pone.0274289.s007.docx]

|  | Imperial College London  Centre for Haematology  Commonwealth Building  Hammersmith Campus  Du Cane Road,  London, W12 0NN, UK  **Nichola Cooper**  Clinical Reader in Immune Haematology  [n.cooper@imperial.ac.uk](mailto:n.cooper@imperial.ac.uk)  Mobile: +447766546432  18^th^ July 2022 |
| --- | --- |
|  |  |

Dear Editor,

**Ref: (PONE-D-22-08475) Malik et al., ‘Distorted TCR repertoires define multisystem inflammatory syndrome in children’**

Many thanks for your recent consideration of this article intended for publication in *PLoS ONE*, and for helpful comments that have been raised by the reviewer.

We have addressed all the comments and believe the manuscript to be significantly strengthened as a result. Our specific responses to individual comments are set out in detail below. We believe the data presented could lead to the development of more specific test for early identification of multisystem inflammatory syndrome in children enabling physicians to prevent the development of severe, life-threatening complications in children who are infected with SARS-CoV-2.

The funders had no role in study design, data collection and analysis, decision to publish, or preparation of the manuscript. E.N.T., M.S.T. and J.H. have a financial interest in Etcembly Ltd. The other authors declare no competing interest. This does not alter our adherence to PLOS ONE policies on sharing data and materials

Many thanks for your consideration and we look forward to hearing back from you.

Sincerely,

Dr Nichola Cooper MA, MD, MBBS, FRCP, FRCPath

Clinical Reader in Immune Haematology and Honorary Consultant Haematologist

*Journal’s comments:*

1. *Please ensure that your manuscript meets PLOS ONE's style requirements,*

*including those for file naming.*

Response: We have now changed the style requirements.

2. *Please amend your current ethics statement to address the following concerns: Did participants provide their written or verbal informed consent to participate in this study? b) If consent was verbal, please explain i) why written consent was not obtained, ii) how you documented participant consent, and iii) whether the ethics committees/IRB approved this consent procedure.*

Response: Written consent was obtained. We have changed our ethics statement.

3. *We note that the grant information you provided in the ‘Funding Information’ and ‘Financial Disclosure’ sections do not match. When you resubmit, please ensure that you provide the correct grant numbers for the awards you received for your study in the ‘Funding Information’ section.*

Response: We have now corrected this.

4. *Thank you for stating the following financial disclosure: N.C and M.D.V. are partially funded by Imperial College NIHR BRC. A.M. is funded by the Jon Moulton Charity.” Please state what role the funders took in the study.  If the funders had no role, please state: "The funders had no role in study design, data collection and analysis, decision to publish, or preparation of the manuscript." If this statement is not correct you must amend it as needed. Please include this amended Role of Funder statement in your cover letter; we will change the online submission form on your behalf.*

Response: The funders had no role in study design, data collection and analysis, decision to publish, or preparation of the manuscript.

5. *Thank you for stating the following in the Competing Interests section: “E.N.T., M.S.T. and J.H. have a financial interest in Etcembly Ltd. The other authors declare no competing interest.” Please confirm that this does not alter your adherence to all PLOS ONE policies on sharing data and materials, by including the following statement: "This does not alter our adherence to PLOS ONE policies on sharing data and materials.” (as detailed online in our guide for authors* [*http://journals.plos.org/plosone/s/competing-interests*](http://journals.plos.org/plosone/s/competing-interests)*).  If there are restrictions on sharing of data and/or materials, please state these. Please note that we cannot proceed with consideration of your article until this information has been declared. Please include your updated Competing Interests statement in your cover letter; we will change the online submission form on your behalf.*

Response: We confirm that that this does not alter your adherence to all PLOS ONE policies on sharing data and materials, by including the above statement and have changed the cover letter accordingly.

6. *Please include your full ethics statement in the ‘Methods’ section of your manuscript file. In your statement, please include the full name of the IRB or ethics committee who approved or waived your study, as well as whether or not you obtained informed written or verbal consent. If consent was waived for your study, please include this information in your statement as well.*

Response: We have included full ethics statement in the Methods section of our manuscript file.

7. *We note that you have stated that you will provide repository information for your data at acceptance. Should your manuscript be accepted for publication, we will hold it until you provide the relevant accession numbers or DOIs necessary to access your data. If you wish to make changes to your Data Availability statement, please describe these changes in your cover letter and we will update your Data Availability statement to reflect the information you provide.*

Response: We will provide repository information

8. *Please include captions for your Supporting Information files at the end of your manuscript, and update any in-text citations to match accordingly. Please see our Supporting Information guidelines for more information:* [*http://journals.plos.org/plosone/s/supporting-information*](http://journals.plos.org/plosone/s/supporting-information)

*[Note: HTML markup is below. Please do not edit.]*

Response: We have now included the captions for our supporting information files at the end of our manuscript and updatd any in-text citations to match accordingly

*Comments from Reviewer #1:*

1. *In the manuscript the authors state that “whether the specific T cell clones contribute to the hyperinflammatory state or if there is a difference in the T cell repertoire composition, antigen specificity is unknown”. This is not correct, and several studies have covered the repertoire of MIS-C patients on largest cohorts (Moreews et al. Science Immunol 2021, Hoste et al. JEM 2022, Sacco et al Nat Med 2022).* Response: All these papers and other relevant papers have now been cited.
2. *The group of severity are poorly described and the “mild disease” comprise asymptomatic patients (that can thus not be defined as mild) and the severe group only comprise MIS-C patients (no other subset of ICU and pediatric COVID-19). This should be corrected, and the authors should clarify that they compare MIS-C to SARS-CoV2-positive children. The severe group is actually restricted to MIS-C. I suggest to avoid to name this group Severe COVID-19 (MIS-C instead). There are two severe manifestations of COVID-19 in children:
   -Severe / fatal pneumonia, occurring in immunocompromised children (IFN-I pathway, in particular) and MIS-C. Here the authors only highlight the MIS-C phenotype.*
   Response: We have now changed the text in the manuscript to represent this.
3. *The use of DNA instead of RNA for the TCR sequencing is more challenging and associate a -greater number of biases.*

Response: Although using DNA is more challenging and can lead to greater number of biases, the protocol we employed addresses these issues. The protocol used in this study has been standardised and used routinely in clinical setting for minimal residual disease (MRD) in cancer (Bartram et al.). Furthermore, analysis of the raw TCR sequences was performed using MiXCR which removes PCR errors before assembling clonotypes. Using DNA for bulk sequencing is also more quantitative, because there is one copy/cell, so the result will look more similar to e.g. single-cell sequencing, as opposed to RNA sequencing where you have varying number of RNA copies/cell.

4. *The junctional diversity of TRBV11-2 was also previously reported. In addition, it is present in both CD4/CD8 T cells and some functional studies have also tested T cell activation to various SARS-CoV2 antigens in MIS-C patients (Moreews et al. Hoste et al…).*
   Response: We have now added these references to the manuscript.
5. *Actually the polyclonal expansion of T cells is a feature of superantigen immune reaction that was highlight in previous (omitted)studies. The other patients that experience an acute infection present an antigen-specific immunity that is also reported and physiological.*

Response: We have added the relevant references in the manuscript.

6. *The authors state that the TCR bias is responsible for the hyperinflammatory syndrome but this causal link is not supported by any data provided here.*
   Response: We agree with the reviewer and have changed the wording in the manuscript to represent that this is a speculation.

*The timing to infection should be indicated. The so-called group “mild “ was possibly sampled at the acute phasis of the infection whereas the MIS-C are sampled at the time of the episode which is post-infectious (about 4 weeks after the virus encounter). This information is important.*
Response: There was no significant difference in sampling between the two groups. The mild group was sampled at a median of 8 days post COVID-19 diagnosis and the MIS-C group was sampled at a median of 10 days post COVID-19 diagnosis.

7. *The clinical data on the MIS-C are missing (vasoplegia, blood pressure, shock, erythema…).
   MIS-C usually occurs in patients with no comorbidities. How MIS-C was confirmed in the patient with hepatoblastoma ?*
   Response:  Patient with hepatoblastoma  was diagnosed based on having central nervous system disease, a clinical manifestation of MIS-C. We don’t have access to any further clinical data on the patients.
8. *The TRBV11-2/ Vb21.3 expansion is now considered as a diagnosis marker. How was individual TRBV11-2 expansion (especially in the patient with hepatoblastoma)?*
   Response:  Patient with hepatoblastoma was diagnosed based on having central nervous system disease, a clinical manifestation of MIS-C. We don’t have access to any further clinical data on the patients. The spread of TRBV-12 expansion is shown on the box plot. The level of TRBV11-2 is significantly higher in MIS-C compared to controls; therefore, it is possible to say that it can be used as a biomarker. The spread in the MIS-C patients was quite even.
9. *Is there any pediatric controls in the repertoire assessment?*
   Response:  There are paediatric control included in the repertoire analysis and referred to as ‘pre-COVID-19’ or ‘unexposed’ in the figures.
10. *In total, this is a replication study of numerous previous work on MIS-C (not severe pediatric COVID) and use a more challenging technique (DNA seq) to address this question on a lowest number of patients. The add-value of this work is limited compared to previous studies and the authors may provide information on how this report replicate previous work and what is new.*
    Response: Although the expansion and the polyclonality of TRBV11-2 have been shown before in MIS-C, we however, report the use of two metrics to define MIS-C in children infected with SARS-CoV-2: 1) CDR3-independent expansion of TRBV11-2+ T cells, 2) a lack of SARS-CoV-2 specificity in TRBV11-2+ T cells, measured by distance to Sars-Cov2-specific MIRA clones. These two metrics can serve as biomarkers for early detection of MIS-C guiding physicians to start precision immunotherapeutics that can prevent the development of severe, life-threatening complications and lasting disability in children. More specifically, the use of network embedding and the distance to MIRA analysis is the added value of our study.
